# Vascular inflammation in chronic kidney disease: the role of uremic toxins in macrophage activation

**DOI:** 10.3389/fcvm.2025.1574489

**Published:** 2025-03-25

**Authors:** Prabhash Kumar Jha, Toshiaki Nakano, Lucas Yuji Umesaki Itto, Miguel Cantadori Barbeiro, Adrien Lupieri, Elena Aikawa, Masanori Aikawa

**Affiliations:** ^1^ Center for Excellence in Vascular Biology, Brigham and Women’s Hospital, Harvard Medical School, Boston, MA, United States; ^2^ Department of Medicine and Clinical Science, Graduate School of Medical Sciences, Kyushu University, Fukuoka, Japan; ^3^ Center for Interdisciplinary Cardiovascular Sciences, Brigham and Women’s Hospital, Harvard Medical School, Boston, MA, United States; ^4^ Channing Division of Network Medicine, Brigham and Women’s Hospital, Harvard Medical School, Boston, MA, United States

**Keywords:** chronic kidney disease (CKD), inflammation, vascular disease, uremic toxins, indoxyl sulfate, macrophages, cardiovascular risk, end-stage renal disease (ESRD)

## Abstract

Chronic kidney disease (CKD) is a progressive condition characterized by the gradual loss of kidney function, leading to the accumulation of uremic toxins in the bloodstream. These toxins play a pivotal role in mediating vascular inflammation, a key contributor to the high cardiovascular morbidity and mortality observed in CKD patients. This review article explores the intricate mechanisms by which uremic toxins accelerate vascular inflammation. Macrophages, as versatile immune cells, are central to the inflammatory response. Evidence suggests that the uremic milieu influences macrophage biology. In this review article, we focus on the signaling through which uremic toxins, particularly indoxyl sulfate—an independent risk factor for cardiovascular complications in CKD patients, modulate macrophage activation and function, and how these changes contribute to vascular inflammation, leading to the increased cardiovascular risk. Investigation of such mechanisms provide molecular bases for the development of new therapies that retard the development of cardiovascular disorders in CKD patients.

## Introduction

Chronic kidney disease (CKD) is one of the most significant global health burden of the 21st century, in part due to the rise in risk factors such as diabetes, hypertension, dyslipidemia and obesity (1). Chronic Kidney Disease (CKD) affects over 800 million people worldwide, impacting 8%–16% of adults. It also significantly increases the risk of cardiovascular disease (CVD), with the prognosis for CVD in CKD patients being particularly poor (2). The data from Canada showed the life expectancy of 55-year-old end-stage renal disease (ESRD) patients is only 5.6 years (3). A major cause of death in ESRD worldwide is CVD (40%) and this population is growing (4, 5). The incidence of myocardial infarction and stroke is 5–15-fold higher and cardiovascular mortality is 30 times higher in the dialysis patients than in the general population (4, 6, 7). Studies revealed a higher prevalence of atherosclerotic lesions in CKD (8–10). However, underlying mechanisms for accelerated atherogenesis in CKD remain unclear and effective medical solutions are limited. Prior research by our group (11–14, 15) and others (16–22) reported that CKD aggravates major signs of vascular disorders, such as inflammation and calcification, in clinical and experimental atherosclerosis.

Pro-inflammatory activation of macrophages plays a pivotal role in chronic inflammation and accelerates vascular diseases (23). Evidence suggests macrophages are a group of cells with heterogeneous phenotypes (24–27). Different sets of signaling pathways may lead to changes in macrophage phenotypes towards pro-inflammatory subsets. For example, we reported that Delta-like ligand 4 (Dll4) of the Notch pathway promotes pro-inflammatory activation (28) probably by regulating macrophage heterogeneity (14). Notch signaling determines the growth, differentiation, and survival of various cell types in diverse tissues (29, 30). Our studies demonstrated that the Dll4-Notch axis promotes vascular and metabolic disorders (14, 31).

Persistent inflammation in CKD, which significantly contributes to vascular complications, may represent sustained activation of macrophages in CKD. Among the various immune cells involved, macrophages appear to play a pivotal role in mediating vascular inflammation in CKD (14, 16). Macrophages are highly plastic cells that can adopt different functional states in response to microenvironmental signals. A traditional concept classified macrophages into two phenotypes: pro-inflammatory macrophages and anti-inflammatory macrophages (e.g., M1 vs. M2 phenotypes) (32, 33), while recent current understandings suggest a more complex, multi-dimensional heterogeneity (26, 27).

Uremic toxins, byproducts of impaired renal excretion, significantly contribute to the pathogenesis of cardiovascular and metabolic disorders in CKD. Uremic toxins in CKD play a crucial role in macrophage activation and vascular inflammation, comparable to traditional CVD risk factors such as hypertension, diabetes, and dyslipidemia. Their accumulation due to impaired kidney function triggers systemic inflammation, which worsens as CKD progresses. From stage 3 CKD (eGFR <60 ml/min/1.73 m^2^) onward, uremic toxin-induced inflammation becomes significant, escalating in stages 4 and 5, where heightened toxin levels further intensify inflammatory responses and cardiovascular risk (34, 35). Among these, indoxyl sulfate, a protein-bound uremic toxin (36) derived from dietary tryptophan metabolism—has garnered considerable clinical attention in cohorts of humans (2, 4, 7, 37, 38). Elevated indoxyl sulfate levels are strongly associated with vascular dysfunction, endothelial cell activation, and accelerated atherosclerosis, highlighting its pivotal role in exacerbating CKD-related cardiovascular morbidity. Its dual impact on oxidative stress and pro-inflammatory pathways underscores the need for mechanistic studies to unravel its contribution to vascular complications, paving the way for targeted therapeutic interventions.

## Role of macrophages in CKD-associated vascular inflammation

Macrophages play a crucial role in the pathophysiology of CKD, particularly in driving vascular inflammation, which is a hallmark of CKD-related cardiovascular complications. The activation of macrophages in CKD occurs through various mechanisms that are influenced by metabolic dysregulation, oxidative stress, and immune signaling.

Macrophages, as key players in innate immunity, contribute significantly to inflammation and tissue remodeling in CKD. In the context of CKD, there is an increase in pro-inflammatory stimuli that activates macrophages, skewing them towards a pro-inflammatory phenotype. These macrophages secrete high levels of pro-inflammatory cytokines such as TNF-α, IL-1β, and IL-6, which propagate inflammation and damage to the vascular endothelium (39). This persistent macrophage-mediated inflammation not only promotes endothelial dysfunction but also contributes to vascular calcification, arterial stiffening, and the progression of atherosclerosis in CKD (40, 41). Moreover, macrophages also play a role in the recruitment and activation of other immune cells, such as T cells, that exacerbate vascular inflammation. Activated macrophages release chemokines, including MCP-1, that attract monocytes to the sites of injury, perpetuating a cycle of inflammation and vascular damage (42).

**Figure 1:**
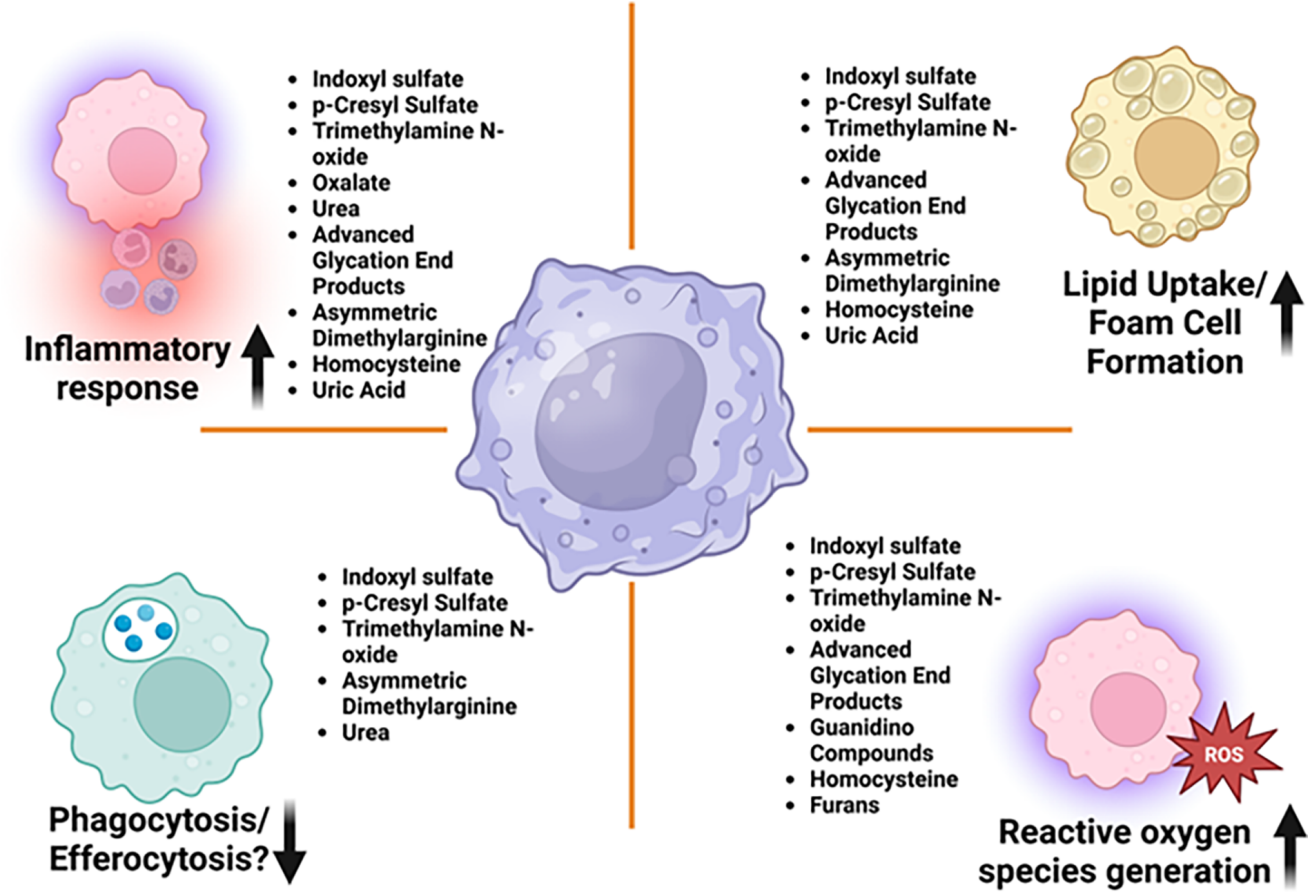
Uremic toxins affecting key macrophage functions leading to vascular inflammation in CKD—macrophages are central to both the development and progression of vascular inflammation. Their roles are diverse, encompassing lipid uptake, foam cell formation, cytokine release, and the regulation of plaque stability or destabilization. Various uremic toxins have been shown to impair macrophage function, thereby promoting the progression of chronic kidney disease and its associated cardiovascular complications, as illustrated in this figure.

Accumulation of uremic toxins in CKD contributes to macrophage activation. In cultured human primary macrophages, these toxins induce oxidative stress and enhance the secretion of pro-inflammatory cytokines by macrophages (34). This oxidative environment shifts macrophages towards a pro-inflammatory phenotype, exacerbating vascular inflammation (43). As depicted in Figure 1, several mechanisms mediated by uremic toxins underlie macrophage activation in CKD, leading to vascular inflammation:

1. **Oxidative Stress:** CKD is characterized by heightened oxidative stress due to reduced renal function and impaired clearance of reactive oxygen species (ROS). The elevated oxidative stress activates macrophages through redox-sensitive pathways, such as the activation of NF-κB, which promotes the production of pro-inflammatory mediators (44). These macrophages further release ROS, amplifying the local oxidative stress and contributing to endothelial dysfunction and vascular inflammation in cultured vascular smooth muscle cell (45).
2. **Chronic Inflammation:** CKD is a state of chronic low-grade inflammation. This inflammatory milieu, characterized by elevated levels of cytokines such as IL-6 and CRP, provides continuous activation signals to macrophages (46). Chronic inflammation drives macrophage polarization towards the M1 phenotype, increasing the release of inflammatory cytokines that perpetuate vascular injury (16).
3. **Dyslipidemia:** CKD is often associated with dyslipidemia, which contributes to the activation of macrophages. Lipoproteins, particularly oxidized LDL, can be internalized by macrophages, leading to foam cell formation. In cultured peritoneal macrophages isolated from apoE−/− mice, this process not only promotes atherosclerosis but also triggers macrophage activation, enhancing the release of pro-inflammatory cytokines and contributing to vascular inflammation (47).
4. **Phagocytosis:** In patients with CKD, the accumulation of uremic toxins such as indoxyl sulfate impairs macrophage functions, including phagocytosis (48) and efferocytosis. This dysfunction primarily stems from heightened oxidative stress and disrupted intracellular signaling, which collectively compromise the ability of macrophages to effectively clear apoptotic cells and pathogens. Such impairments drive persistent inflammation, further accelerating CKD progression. Notably, targeting these dysregulated pathways holds therapeutic promise for restoring immune function in CKD. For example, indoxyl sulfate has been shown to enhance macrophage responses to lipopolysaccharide (LPS), resulting in elevated production of ROS and pro-inflammatory cytokines. Furthermore, evidence suggests that indoxyl sulfate directly suppresses macrophage phagocytic activity through oxidative mechanisms mediated by pathways such as NADH oxidase (NOX), protein kinase C (PKC), and phosphoinositide 3-kinase (PI3K) (49). The role of uremic toxins in macrophage efferocytosis within the context of CKD is an underexplored area of research that holds significant promise for future studies.

## Signaling pathways involved in macrophage activation in CKD-associated vascular inflammation

Macrophages are pivotal contributors to vascular inflammation in CKD, driven by various signaling pathways that regulate their activation. Understanding these pathways is essential to grasp the mechanisms underlying macrophage-induced vascular injury in CKD. The major signaling pathways implicated in macrophage activation in CKD are discussed below.

**Figure 2:**
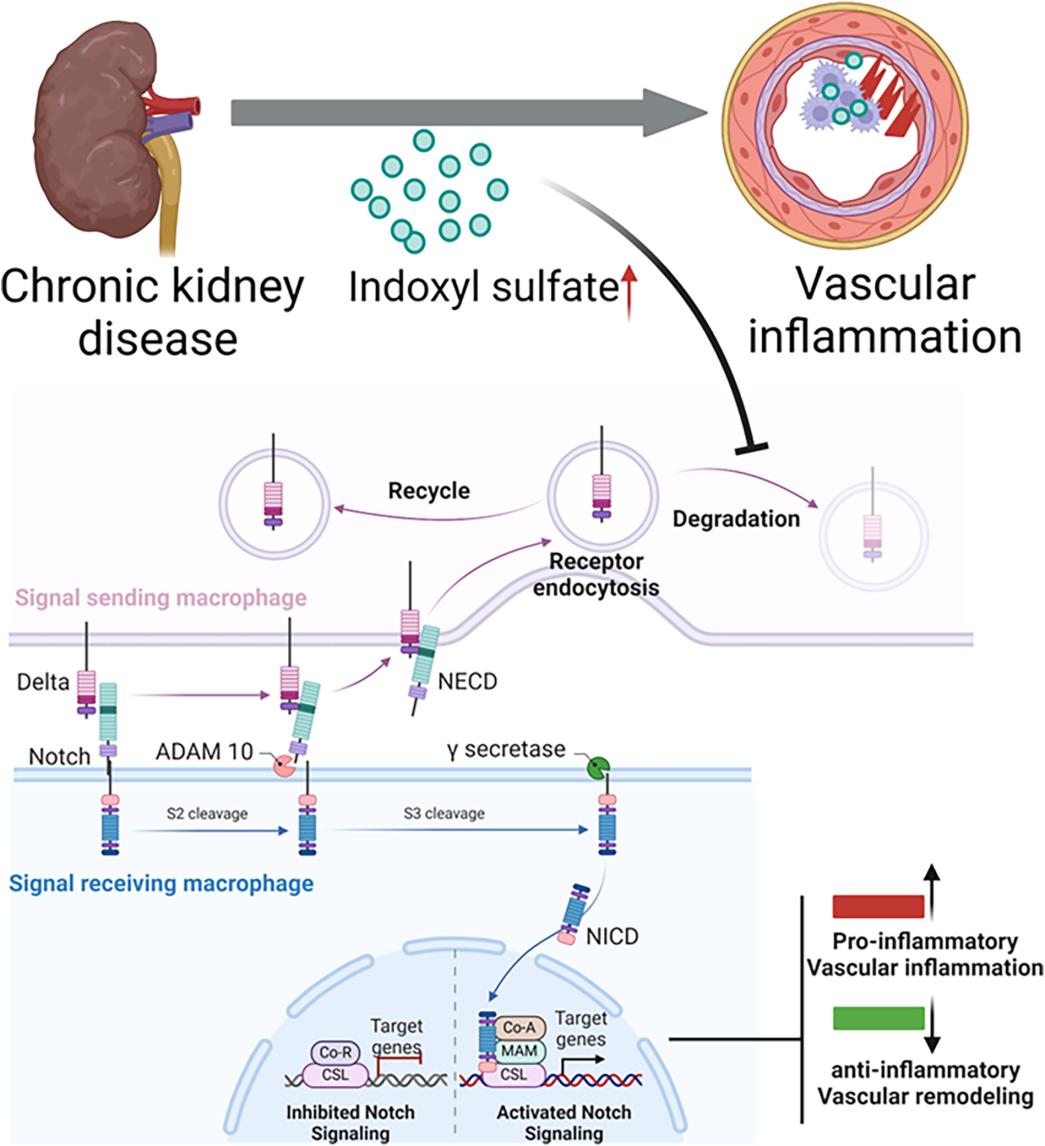
Proposed mechanism of indoxyl sulfate-mediated proinflammatory activation of macrophages, contributing to vascular inflammation in chronic kidney disease. lndoxyl sulfate, a uremic toxin that accumulates in patients with chronic kidney disease undergoing hemodialysis, is taken up by macrophages. Normally, the Dll4 protein undergoes endocytosis and recycling. However, indoxyl sulfate inhibits Dll4 degradation, increasing its recycling. The enhanced interaction between Dll4 and its receptor (e.g., Notch 1) may amplify Notch signaling, driving the expression of proinflammatory genes. This continuous receptor recycling in the signal-sending macrophage promotes a proinflammatory state, ultimately leading to vascular inflammation.

1. **Dll4-Notch signaling:** Notch signaling regulates the mechanism of signal transduction in embryonic development and differentiation of various cell types and organs (29, 30). The Notch pathway in mammals involves five ligands (Jagged1, Jagged2, Dll1, Dll3, and Dll4) and four receptors (Notch1, Notch2, Notch3, and Notch4) (Figure 2). Notch signaling is typically triggered by direct cell-to-cell contact. Notch receptor activation is then regulated by the receptor cleavage. ADAM metalloprotease and γ-secretase mediate this cleavage to release the Notch intracellular domain (NICD). NICD translocate to the nucleus, binds with the transcriptional repressor RBP-Jκ, and leads to transcriptional activation (30, 50).
  Our study by Nakano *et al* (14) demonstrated potential mechanism for pro-inflammatory activation of macrophages in CKD involves crosstalk between OATP2B1 and the Dll4-Notch axis in primary human macrophages. Indoxyl sulfate, taken up by macrophages *via* OATP2B1, may inhibit Dll4 degradation through USP5, leading to increased Dll4 recycling and enhanced Notch signaling, which induces pro-inflammatory gene expression, although other mechanisms may also be involved. Figure 2 demonstrates the proposed mechanism of indoxyl sulfate-mediated proinflammatory activation of macrophages, contributing to vascular inflammation in CKD *via* Dll4-Notch signaling (14).
2. **NF-κB Pathway:** The nuclear factor-kappa B (NF-κB) pathway is a central regulator of macrophage activation in CKD. Uremic toxins such as indoxyl sulfate, which accumulate due to reduced renal clearance, stimulate the NF-κB pathway, leading to increased transcription of pro-inflammatory cytokines such as TNF-α, IL-1β, and IL-6. Upon stimulation by these uremic toxins or other inflammatory signals like advanced glycation end-products (AGEs), NF-κB translocate into the nucleus, where it activates genes involved in macrophage pro-inflammatory responses. The continuous activation of NF-κB in macrophages contributes to a chronic inflammatory state in the vascular endothelium, promoting endothelial dysfunction, vascular calcification, and atherosclerosis in CKD (44).
3. **JAK/STAT Pathway:** The Janus kinase/signal transducer and activator of transcription (JAK/STAT) pathway plays a significant role in the polarization of macrophages towards the pro-inflammatory M1 phenotype in CKD. Activation of this pathway occurs in response to cytokines such as IFN-γ and IL-6, which are elevated in CKD. These cytokines activate JAKs, which in turn phosphorylate STAT proteins, leading to their dimerization and nuclear translocation, where they initiate the transcription of pro-inflammatory genes (51). In preclinical mouse model of CKD, the persistent activation of the JAK/STAT pathway by inflammatory stimuli amplifies macrophage-driven vascular inflammation, contributing to endothelial injury and vascular stiffening (52).
4. **NLRP3 Inflammasome Pathway:** The NLRP3 (NOD-like receptor pyrin domain-containing 3) inflammasome is a crucial intracellular sensor of stress signals, including oxidative stress and metabolic dysregulation in CKD. Activation of the NLRP3 inflammasome in macrophages leads to the cleavage of pro-IL-1β and pro-IL-18 into their active forms, IL-1β and IL-18, which are potent drivers of inflammation (53). Uremic toxins, oxidative stress, and mitochondrial dysfunction seen in CKD contribute to the activation of the NLRP3 inflammasome, promoting macrophage-mediated inflammatory responses and subsequent vascular injury. This pathway has been shown to accelerate atherosclerosis and vascular calcification by enhancing the pro-inflammatory activity of macrophages (54, 55).
5. **MAPK Pathway:** The mitogen-activated protein kinase (MAPK) pathway, comprising ERK, JNK, and p38 MAPK, is another important signaling cascade involved in macrophage activation in CKD. The MAPK pathway is activated in response to various stimuli, including pro-inflammatory cytokines, oxidative stress, and mechanical stress within the vascular wall. Activation of MAPKs in macrophages leads to the production of pro-inflammatory cytokines, such as TNF-α and IL-6, and induces macrophage polarization towards the M1 phenotype. In CKD, the sustained activation of MAPK signaling contributes to vascular inflammation, endothelial dysfunction, and smooth muscle cell calcification, worsening CVD outcomes (56, 57).
6. **TLR Pathway:** Toll-like receptors (TLRs) are key pattern recognition receptors (PRRs) that detect pathogen-associated molecular patterns (PAMPs) and damage-associated molecular patterns (DAMPs). In CKD, the heightened levels of PAMPs and DAMPs, such as AGEs, and oxidative stress by-products, lead to TLR activation, particularly TLR4, in macrophages (58). Upon activation, TLRs initiate downstream signaling through adaptor proteins such as MyD88, resulting in the activation of NF-κB and MAPK pathways, which further enhance the production of pro-inflammatory cytokines. TLR signaling in macrophages is closely linked to vascular inflammation in CKD, promoting endothelial dysfunction and accelerating the development of vascular calcification and atherosclerosis (59).
7. **Aryl Hydrocarbon Receptor (AhR):** The aryl hydrocarbon receptor (AhR) pathway plays a crucial role in macrophage activation in CKD. AhR, a ligand-activated transcription factor, can be activated by various uremic toxins, including indoxyl sulfate, leading to the modulation of macrophage function (60). Activation of AhR influences macrophage differentiation, polarization, and the expression of pro-inflammatory genes, contributing to the chronic inflammation observed in CKD patients (61). Targeting the AhR pathway may offer therapeutic potential for mitigating inflammation and improving outcomes in CKD (60, 62).

Multiple signaling pathways, including NF-κB, JAK/STAT, NLRP3 inflammasome, MAPK, and TLR, are critical in the activation of macrophages in CKD, driving vascular inflammation. These pathways collectively contribute to endothelial dysfunction, vascular calcification, and the progression of atherosclerosis. Understanding these mechanisms provides opportunities to develop targeted therapies aimed at modulating macrophage activation to alleviate vascular inflammation and reduce cardiovascular risk in CKD patients.

## Role of indoxyl sulfate in CKD-associated vascular inflammation

Although CKD patients commonly have CVD risk factors (e.g., hypertension, dyslipidemia, diabetes), such traditional factors cannot entirely explain their increased cardiovascular risk (2, 4, 7, 37, 38). Uremia, defined as the accumulation of solutes, plays a critical role in the pathogenesis of accelerated CVD in CKD, making “uremic” risk factors emerging research spotlights (35, 38, 63). Indoxyl sulfate represents metabolites of dietary tryptophan. After tryptophan's conversion to indole by intestinal bacteria and to indoxyl sulfate in the liver, it is normally cleared by the kidney. When kidney function fails, indoxyl sulfate accumulates in the blood. Among various uremic toxins in CKD, indoxyl sulfate has become prime focus for the following reasons:

1. Clinical and basic research has established that indoxyl sulfate causes cardiovascular organ damage (35, 38, 64–66). While CKD increases various uremic toxins, clinical evidence has established that indoxyl sulfate serves as an independent, powerful risk factor for cardiovascular mortality and adverse cardiovascular events (38, 67, 68), and its levels also associate with the severity of coronary atherosclerosis (67, 69). Indoxyl sulfate induces endothelial dysfunction, through the increased production of NADPH oxidase-derived reactive oxygen species (ROS), the increased expression of intercellular adhesion molecule-1 (ICAM-1) and E-selectin, factors playing key roles in monocyte–endothelial interactions (70, 71). Indoxyl sulfate increases the expression of proinflammatory cytokines, IL-1β, TNF-α, and MCP-1 in macrophages (14, 72, 73). In THP-1 derived macrophages, indoxyl sulfate also decreases the expression of ATP-binding cassette transporter G1 (ABCG1), which is a cholesterol efflux transporter in macrophages, and reduces cholesterol efflux (72). Indoxyl sulfate induces proinflammatory cytokines in human macrophages via the Dll4–Notch signaling pathway (14) and aryl hydrocarbon receptor (AhR) (73), which contribute to atheroma progression. The biology of indoxyl sulfate has thus emerged as an important research topic.
2. Dialysis prolongs the survival of end stage renal disease patients, but cannot completely remove uremic toxins (74). Among gut-derived uremic toxins, indoxyl sulfate and p-cresyl sulfate are largely protein bound in the blood circulation (mostly to albumin) and thus poorly cleared by diffusive dialysis procedures (35, 75).
3. Due to incomplete mechanistic understanding, no effective therapies to reduce levels of indoxyl sulfate are available. In preclinical animal models of CKD, the micro spherical carbon adsorbent AST-120 reduced serum indoxyl sulfate levels, monocyte activation (76), and atherogenesis (6). Small clinical studies showed beneficial effects of AST-120 on carotid intima-media thickness in pre-dialysis patient cohorts (77, 78). Large clinical trials, including a few multicenter, randomized, controlled phase III trials, however, showed no benefits of AST-120 on cardiovascular events in CKD, which may have resulted from the low magnitude of indoxyl sulfate reduction in patients (<10%–20%) (79).

Thus, more mechanistic studies are needed to understand mechanisms by which indoxyl sulfate affects macrophage responses in CKD and to explore strategies to regulate macrophage phenotype in the high indoxyl sulfate milieu.

## Other uremic toxins involved in inflammation in CKD

In addition to indoxyl sulfate, other molecules also participate in pro-inflammatory processes linked to CKD. Evidence has linked uremic toxins other than indoxyl sulfate with cardiovascular morbidity and mortality in patients with CKD. Table 1 depicts comprehensive overview of various uremic toxins implicated in macrophage activation and the resulting vascular inflammation, particularly in the context of CKD.

(1) **P-cresyl sulfate:** Intestinal bacteria metabolize tyrosine and phenylalanine to produce p-cresol, which is taken up and sulfated by SULT1A1 to produce p-cresyl sulfate (pCS) (80, 81). pCS induce oxidative stress, renal fibrosis/inflammation, and an EMT-like process through activation of the RAS pathway (82). In hemodialysis patients, pCS have been associated with an increase in carotid atherosclerotic plaque and is implicated in vascular inflammation, vascular calcification, and atherogenesis (83). pCS induce increased production of ROS, increased expression of NADPH oxidase, and increased expression of pro-inflammatory factors MCP-1 and TNF-α in human endothelial cells and aortic smooth muscle (84). pCS induces osteogenesis in human arterial smooth muscle cells, leading to vascular calcification through oxidative stress and specific signaling pathways (85). pCS can activate macrophages by increasing oxidative burst and phagocytosis. pCS also promote the expression of inflammatory factors and adhesion molecules in macrophages and cultured endothelial cells (86, 87).
(2) **Indole acetic acid:** There are toxins other than indoxyl sulfate derived from the metabolism of tryptophan by the intestinal microbiota with potential for accumulation and toxicity in patients with CKD, such as indole acetic acid. Indole acetic acid has been shown to be an important ligand for the aromatic hydrocarbon receptor (AhR), a ligand-activated transcription factor involved in the expression of enzymes that metabolize xenobiotics, inflammatory cytokines and adhesion molecules, all of which are mediators of inflammation in cardiovascular diseases (88). Patients with advanced CKD had higher levels of indole-3-acetic acid (IAA), which was associated with a significant increase in mortality from cardiovascular events. A previous study demonstrated that IAA increased the expression of endothelial inflammatory genes such as IL-6, IL-8, ICAM-1 and MCP-1 (89).
(3) **Advanced glycation end products:** Advanced glycation end products (AGEs) refer to a diverse group of compounds formed as a result of the non-enzymatic glycation of proteins, lipids and nucleic acids through a complex chain of reactions known as the Maillard reaction (90). Amongst these compounds, N-carboxymethyl lysine, pentosidine and hydroimidazolone are the most extensively studied AGEs and are considered markers of AGE accumulation in tissues (91, 92). The accumulation of AGEs in patients with CKD is a result of oxidative stress and inflammation and can come from external sources such as diet and smoking (93, 94). The kidneys are responsible for filtering and eliminating AGEs, however, AGEs can also become accumulate in the kidneys and cause vascular damage (95). The interaction between AGEs and their receptors, such as product receptors end-to-end glycation, initiate several events that lead to endothelial dysfunction, arterial stiffness, dysregulation of the immune system and progression of atherosclerosis (93).
(4) **Hippuric acid:** Hippuric acid is a protein-bound uremic toxin that is elevated in patients with CKD. Hippuric acid is converted from dietary polyphenols by the gut microbiome into benzoic acid, which is further converted to hippuric acid by glycine-N-acyltransferase in the liver or kidneys (96). Some evidence suggests that the acid hippuric acid contributes to the progression of renal fibrosis and endothelial dysfunction by inducing oxidative stress (35, 97). While specific studies directly linking hippuric acid to macrophage activation are limited, there are some insights into its broader immunomodulatory effects. Hippuric acid has been shown to interact with the GPR109A receptor, which is involved in anti-inflammatory responses. This interaction can potentially influence macrophage activity by promoting anti-inflammatory pathways (98). As a product of gut microbiota metabolism, hippuric acid may contribute to the overall immune modulation by influencing the gut-immune axis. This can indirectly affect macrophage activation and function (99).
(5) **Trimethylamine N-oxide:** Trimethylamine N-oxide (TMAO) is a free water-soluble low molecular weight uremic toxin derived from the intestine (100). Intestinal bacteria produce trimethylamine (TMA) from dietary choline, phosphatidylcholine, L-carnitine and betaine, which is converted into TMAO in the liver by means of flavin-containing monooxygenases (101, 102). TMAO accumulates in the plasma of small cohort of patients with CKD and its concentration correlates with coronary atherosclerosis (103). TMAO has been shown to play a significant role in macrophage activation, particularly in promoting pro-inflammatory responses. TMAO has been found to induce M1 macrophage polarization, which is associated with pro-inflammatory responses. This polarization is mediated through the activation of the NLRP3 inflammasome (104). TMAO enhances the production of pro-inflammatory cytokines such as IL-1β, IL-6, and TNF-α. This is achieved through the activation of the NF-κB pathway, which is a critical regulator of inflammation (105). TMAO can disrupt cholesterol and bile acid metabolism, leading to the formation of foam cells. This process is linked to the upregulation of macrophage scavenger receptors and impaired reverse cholesterol transport (106). Elevated levels of TMAO have been associated with various chronic diseases, including cardiovascular diseases and CKD. The pro-inflammatory effects of TMAO on macrophages contribute to the progression of these conditions (107).
(6) **Visfatin:** Also known as nicotinamide phosphoribosyl transferase (NAMPT) or pre-B colony enhancing factor, is an adipokine primarily, but not exclusively, secreted by visceral adipose tissue. At 52 kDa, it is one of the largest medium-sized molecules to be elevated in uremia (108). Intracellularly, it is involved in the biosynthesis of nicotinamide and adenine dinucleotide, but it is also released extracellularly, where it appears to have a wide range of effects, including stimulating angiogenesis and endothelial cell proliferation. It also promotes vascular smooth muscle cell growth, has anti-apoptotic effects on macrophages, and promotes vascular inflammation and endothelial damage (109). High levels of visfatin expression have been found in atherosclerotic plaques in human studies. In addition, circulating levels of visfatin predict the presence of unstable plaque (110). Visfatin plays a significant role in macrophage activation, particularly in promoting inflammatory responses. Visfatin induces the production of pro-inflammatory cytokines such as IL-1, IL-6, TNF-α, and IL-8 in macrophages. This is mediated through the activation of the NF-κB pathway (111). Visfatin promotes cholesterol accumulation in macrophages by upregulating scavenger receptors like SR-A and CD36. This process contributes to the formation of foam cells, which are crucial in the development of atherosclerosis (112). Visfatin can induce the expression of chemokines such as CCL20, which attract other immune cells to the site of inflammation, thereby amplifying the immune response (111).
(7) **Kynurenic acid:** Kynurenic acid is a metabolite of the kynurenine pathway, plays a significant role in both vascular inflammation and macrophage activation. In cultured Bone marrow-derived macrophages, kynurenic acid suppresses the activation of the NLRP3 inflammasome in macrophages, which reduces the production of pro-inflammatory cytokines such as IL-1β (113). This anti-inflammatory action helps mitigate vascular inflammation. Kynurenic acid acts as a ligand for the AhR, a receptor involved in regulating immune responses. AhR activation by kynurenic acid leads to anti-inflammatory effects, which are beneficial in reducing vascular inflammation (114). Kynurenic acid influences various immune pathways, including NF-κB, which is crucial for the regulation of inflammation and immune responses in macrophages (115). By modulating inflammatory responses, kynurenic acid contributes to the protection against cardiovascular diseases (115).

**Table 1:** A comprehensive overview of various uremic toxins implicated in macrophage activation and the resulting vascular inflammation, particularly in the context of chronic kidney disease. Each toxin is categorized by its biochemical nature, source, and mechanism of action. The table highlights how these toxins influence macrophage behavior and contribute to inflammatory processes in vascular tissues. The referenced studies provide further insights into the role of these uremic toxins in macrophage-mediated inflammation and their broader implications for cardiovascular health in CKD patients.

Uremic Toxin	Category	Source	Mechanism of Action	Effects on Macrophages/Vascular Inflammation	References
Indoxyl sulfate	Protein-bound	Tryptophan metabolism	Inhibits endothelial proliferation, macrophage inflammation and promotes vascular calcification	Enhances macrophage mediated inflammation, increases vascular inflammation	(14, 118)
p-Cresyl sulfate	Protein-bound	Tyrosine and phenylalanine metabolism	Disrupts endothelial function, induces oxidative stress	Promotes vascular inflammation, increases endothelial permeability	(86, 118)
Advanced glycation end products (AGEs)	Protein-bound	Glucose metabolism	Cross-links proteins, induces oxidative stress	Promotes inflammation, enhances macrophage activation	(93, 95)
Phenylacetic acid	Protein-bound	Phenylalanine metabolism	Disrupts cellular function, induces oxidative stress	Increases macrophage activation, promotes vascular inflammation	(119)
Kynurenic acid	Protein-bound	Tryptophan metabolism	Modulates immune response, induces oxidative stress	Enhances macrophage activation, promotes vascular inflammation	(114, 115)
Urea	Unbound	Protein catabolism	Inhibits cellular metabolism, induces oxidative stress	Increases macrophage activation, promotes vascular inflammation	(119)
Creatinine	Unbound	Muscle metabolism	Impairs cellular energy metabolism, induces oxidative stress	Contributes to macrophage and endothelial dysfunction, promotes vascular inflammation	(119)
Asymmetric dimethylarginine (ADMA)	Unbound	Protein metabolism	Inhibits nitric oxide synthesis, induces oxidative stress	Promotes endothelial dysfunction, enhances vascular inflammation	(119)
Trimethylamine-N-oxide (TMAO)	Unbound	Choline metabolism	Disrupts lipid metabolism, induces oxidative stress	Promotes atherosclerosis, promotes macrophage activation and enhances vascular inflammation	(103–105)
Uric acid	Unbound	Purine metabolism	Induces oxidative stress, promotes crystal formation	Enhances macrophage activation, promotes vascular inflammation	(120)
Homocysteine	Protein-bound	Byproduct of methionine metabolism	Causes oxidative stress and endothelial dysfunction	Induces macrophage activation and promotes inflammatory responses	(121, 122)
Methyl guanidine	Unbound	Protein catabolism	Causes oxidative stress and apoptosis	Induces macrophage activation, leading to increased pro-inflammatory cytokine release	(123)
Hippuric acid	Protein-bound	dietary polyphenols	Causes oxidative stress and endothelial dysfunction	unknown	(99)
Visfatin	Protein	secreted by visceral adipose tissue	promotes vascular inflammation and endothelial damage	Cholesterol accumulation in macrophages	(109, 112)

## Future perspectives

The role of uremic toxins in macrophage activation and vascular inflammation provides critical insight into CKD management. Since CVD is a leading cause of morbidity and mortality in CKD patients, understanding the cellular mechanisms underlying vascular inflammation is essential for developing targeted therapies. Therapeutic strategies aimed at reducing uremic toxin levels, such as the use of intestinal adsorbents or gut microbiota modulators (e.g., probiotics), may help mitigate macrophage-driven vascular inflammation. Probiotics have gained significant attention as a natural biotreatment due to their health-promoting effects and potential to combat diseases like CKD (116). The intestinal microbiota has emerged as a key contributor to CKD progression and complications, highlighting the importance of selecting probiotic strains based on specific functional biomarkers. Over the past decade, interest in probiotics for CKD has surged, fueled by their potential to reduce uremic toxin production and enhance renal function, as evidenced by *in vitro*, animal, and human studies (117). However, high-quality clinical trials assessing their therapeutic efficacy in CKD remain limited. Additionally, targeting macrophage activation and polarization, perhaps by modulating upstream regulators of specific signaling mechanisms such as NF-κB or NLRP3, could offer new avenues for reducing vascular complications in CKD. Antioxidants or drugs that reduce ROS generation could also serve as potential treatments by attenuating oxidative stress-induced inflammation.

In conclusion, targeting the macrophage-uremic toxin link in CKD presents a promising strategy for mitigating cardiovascular risks in these patients. Future clinical approaches should prioritize both reducing toxin levels and directly modulating immune responses to curb vascular inflammation. This dual-focus strategy not only emphasizes the significance of managing uremic toxins but also highlights the necessity of personalized interventions to better protect CKD patients from cardiovascular complications.
